# Closure strategies post-EMR for esophageal SMTs

**DOI:** 10.1007/s00464-026-13078-9

**Published:** 2026-07-09

**Authors:** Lingling Chen, Genhua Yang, Wei Gong, Chongju Bao

**Affiliations:** 1 Department of Gastroenterology, Fuzhou Second General Hospital, Fuzhou, 350000 Fujian Province China; 2 Department of Gastroenterology, Shenzhen Hospital of Southern Medical University, No.13 Xinhu Road, Shenzhen, 510086 Guangdong Province China

**Keywords:** Esophageal submucosal tumors, Endoscopic mucosal resection, Wound non-closure, Comparative study, Single-center retrospective analysis

## Abstract

**Background:**

Endoscopic mucosal resection (EMR) has become a widely adopted minimally invasive treatment for esophageal submucosal tumors (SMTs). Although prophylactic clip closure of the post-EMR wound is commonly performed to reduce complications, its clinical necessity and efficacy lack robust evidence. This study therefore aimed to compare the clinical outcomes of clip closure versus non-closure following EMR for esophageal SMTs.

**Methods:**

This retrospective, single-center cohort study reviewed consecutive patients who underwent EMR for esophageal SMTs between January 2022 and November 2025. Based on post-EMR wound management, patients were allocated to a closure group (prophylactic metallic clip closure) and a non-closure group (observation only). Patient demographics, lesion characteristics, pathological diagnoses, and clinical outcomes including postoperative complications (bleeding, perforation), hospital stay, and costs were collected and analyzed.

**Results:**

A total of 84 patients (closure: *n* = 52; non-closure: *n* = 32) were included in the analysis. The two groups were well-balanced regarding baseline patient and lesion characteristics, with most tumors being small (≤ 10 mm), superficial (originating from the muscularis mucosae), and histologically confirmed as leiomyomas. The complication rate was minimal, with only one minor, conservatively managed bleeding event recorded in the closure group (1.9% vs. 0%; *P* = 1.0) and no perforations in either cohort. Similarly, no significant intergroup differences were observed for postoperative hospital stay (3.98 ± 1.18 vs. 4.47 ± 1.19 days; *P* = 0.07) or total hospitalization costs (9745.08 ± 3123.66 vs. 9180.34 ± 2647.80 yuan; *P* = 0.397).

**Conclusions:**

For small esophageal SMTs (≤ 10 mm), prophylactic clip closure after EMR does not confer significant advantages over non-closure in reducing complications, hospital stay, or costs. Therefore, its routine use is not warranted.

Esophageal submucosal tumors (SMTs) are lesions originating from tissues beneath the mucosal layer, including the muscularis mucosa, submucosa, or muscularis propria [1–3]_._ They encompass various pathological types, such as leiomyomas, stromal tumors, cysts, and others [4–6]_._ Leiomyoma is the most common type [7, 8]_._ These lesions are typically discovered incidentally during endoscopic examinations [9, 10]_._

With advancements in endoscopic techniques, endoscopic mucosal resection (EMR) has become a widely adopted minimally invasive approach for treating superficial and well-defined esophageal SMTs [11–14]. Compared with traditional surgical methods, EMR offers advantages such as minimal trauma, faster recovery, and shorter hospital stays. It enables complete lesion removal and provides pathological specimens, which are crucial for definitive diagnosis and guiding subsequent management.

However, the artificial ulcers created by EMR are associated with complications, including bleeding, perforation, and delayed healing [15, 16]_._ Due to the relatively thin esophageal wall, optimal wound management is critical to minimizing adverse events, particularly after resection of larger lesions or those in challenging locations. In recent years, endoscopic metallic clip closure techniques have been widely adopted to achieve hemostasis and facilitate wound closure in various gastrointestinal lesions. Following EMR, proactive closure of the wound using metallic clips can theoretically approximate wound edges, reduce the exposed area, and provide hemostatic compression, thereby potentially lowering the risk of bleeding and perforation and accelerating mucosal healing [17–20]_._

The clinical necessity, efficacy, and safety of routine prophylactic clip closure following EMR for esophageal SMTs remain inadequately defined due to a paucity of high-level evidence. Current literature largely comprises studies on clip closure after endoscopic resections in the stomach and colorectum, with limited and sporadic data on esophageal lesions [21–25]_._ Systematic evaluations specifically addressing this clinical scenario (EMR for esophageal SMTs) remain scarce. In clinical practice, physicians’ decisions are often based on personal experience and institutional protocols, highlighting an urgent need to clarify the indications, technical standards, and long-term impact on patient outcomes.

Therefore, this retrospective study was conducted to systematically compare outcomes between patients undergoing prophylactic clip closure and those not undergoing closure after EMR for esophageal SMTs. We specifically assessed postoperative complications (including immediate or delayed bleeding and perforation), hospital stay duration, medical costs, and long-term follow-up outcomes. This study aims to evaluate the practical effectiveness of prophylactic metallic clip closure in this setting, with the goal of providing an objective basis for optimizing postoperative management strategies. Ultimately, these findings may contribute to more standardized clinical protocols, supporting individualized treatment and enhancing patient care.

## Materials and methods

### Study population

This was a retrospective cohort study conducted at a single center. Consecutive patients diagnosed with esophageal SMTs who underwent EMR at Shenzhen Hospital of Southern Medical University between January 2022 and November 2025 were identified and included using the electronic medical record system. This study was reviewed and approved by the Medical Ethics Committee of Shenzhen Hospital of Southern Medical University (Approval No. NYSZYYEC2026K025R001). All procedures performed in studies involving human participants were in accordance with the ethical standards of the institutional research committee.

### Inclusion criteria

1. Initial diagnosis of esophageal SMTs by white-light endoscopy before surgery;
2. Underwent EMR treatment and obtained complete pathological diagnosis;
3. Complete clinical data.

### Exclusion criteria

1. Incomplete endoscopic resection or postoperative pathology confirming malignancy;
2. Contraindications to endoscopic treatment;
3. Severe coagulation dysfunction or failure of vital organ function;
4. Incomplete medical records.

Based on the management of the post-EMR wound, patients were allocated to two groups: the closure group (active wound closure using metal clips after EMR) and the non-closure group (no mechanical closure of the wound postoperatively, only routine observation). Ultimately, a total of 84 patients were included in the analysis, with 52 in the closure group and 32 in the non-closure group.

### Surgical equipment and procedure

**Fig. 1:**
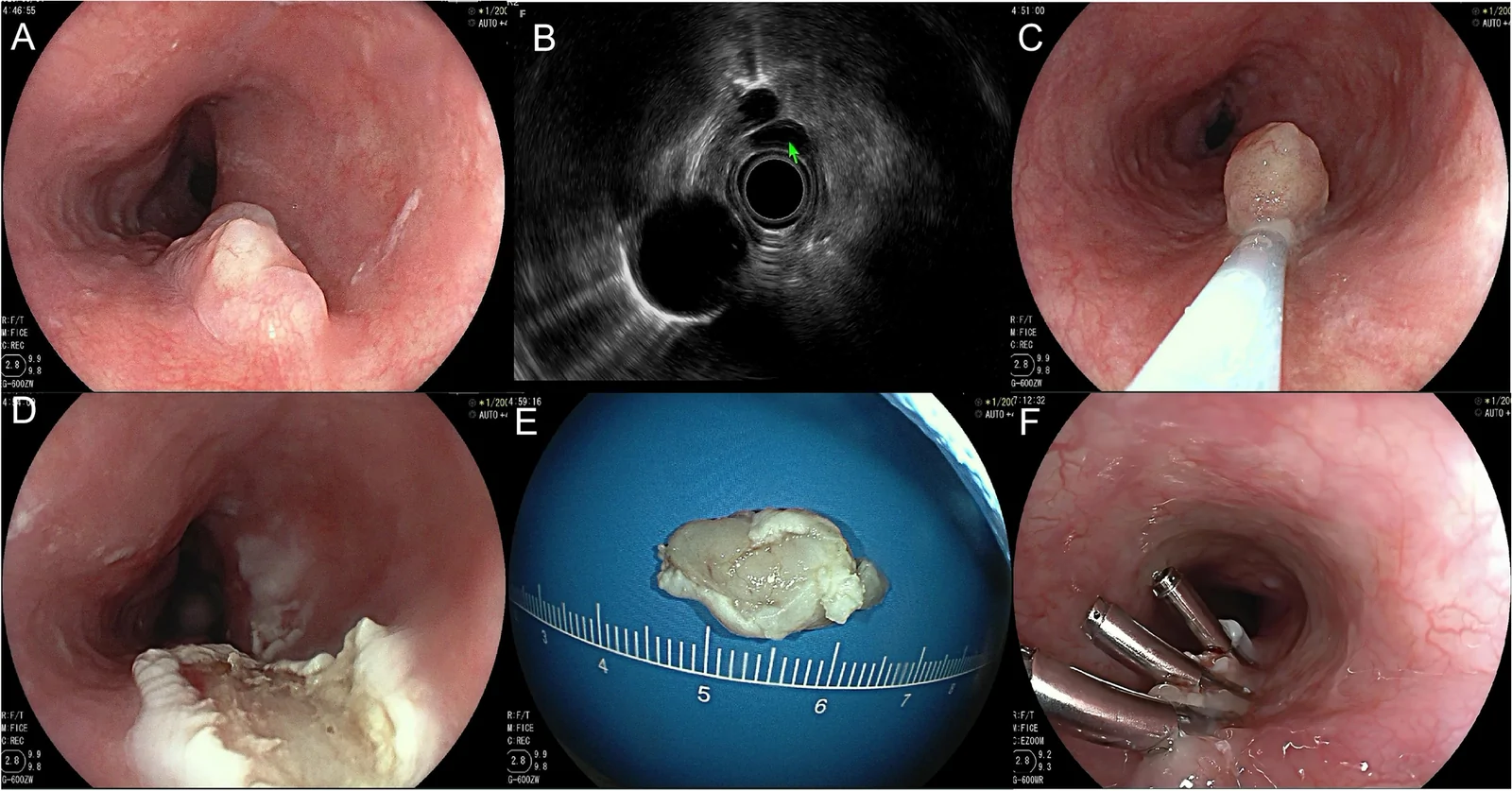
Representative case of endoscopic mucosal resection (EMR) for esophageal submucosal tumors (SMTs). **A** White light endoscopy revealed a 15 mm × 8 mm submucosal lesion in the mid-esophagus. **B** Endoscopic ultrasound showed the lesion originated from the muscularis mucosa. **C** The lesion was resected by EMR. **D** The mucosal wound after EMR. **E** Specimen of esophageal submucosal tumor. **F** Closure of the defect with metallic clips

All procedures were performed by experienced, board-certified endoscopists. Operations were conducted under intravenous anesthesia using high-definition endoscopes and standard EMR instruments. The EMR procedure followed the standard protocol: after submucosal injection of normal saline (containing a small amount of indigo carmine dye), the lesion was completely resected using a snare (Fig. 1A–E). After the procedure, the operator decided whether to close the wound based on intraoperative conditions (including wound size, depth, location, and bleeding tendency) and personal experience. In the closure group, prophylactic metallic clips were applied to approximate and secure the wound edges (Fig. 1F). In the non-closure group, the wound was left to heal by secondary intention without clip application.

### Data collection and analysis

The following data were collected through the hospital information system:

1. Baseline patient characteristics: gender, age.
2. Lesion characteristics: tumor location (upper esophagus: 15–24 cm from the incisors; middle esophagus: 24–32 cm from the incisors, and lower esophagus: 32–40 cm from the incisors), maximum diameter (≤ 10 mm or > 10 mm), and layer of origin assessed by endoscopic ultrasound (EUS).
3. Perioperative data: postoperative pathological results, length of hospital stay, total hospitalization cost, postoperative diet, and postoperative treatment regimens.
4. Primary outcomes: postoperative complications, including bleeding and perforation.

### Statistical methods

Statistical analysis was performed using SPSS software version 25.0. If significant imbalances in baseline characteristics were identified between the two groups, propensity score matching can be performed. Continuous variables with a normal distribution were expressed as mean ± standard deviation and compared between groups using the independent-samples t-test. Continuous variables that were not normally distributed were presented as median (interquartile range) and compared using the Mann–Whitney U test. Categorical variables were presented as counts and percentages, and comparisons between groups were performed using the chi-square test or Fisher’s exact test, as appropriate. A *P* < 0.05 was considered statistically significant.

## Results

### Baseline characteristics of patients

A total of 84 patients with esophageal SMTs who underwent EMR treatment were included in this study. Based on the postoperative wound management method, patients were allocated to a closure group (*n* = 52) and a non-closure group (*n* = 32). There were no statistically significant differences in baseline characteristics such as age and gender between the two groups (all *P* > 0.05), demonstrating that the groups were comparable at baseline (Table 1).

**Table 1:** Comparison of baseline characteristics between the two groups

Characteristic	Closure group (*n* = 52)	Non-closure group (*n* = 32)	*P*-value
Gender (*n*, %)			
Male	29 (55.8%)	22 (68.8%)	0.237
Female	23 (44.2%)	10 (31.2%)	
Age (years) (mean ± SD)	49.83 ± 11.50	52.90 ± 12.99	0.265

### Lesion characteristics

#### Location distribution

Based on the distance from the incisors, lesions were categorized as upper esophagus (15–24 cm from the incisors), middle esophagus (24–32 cm from the incisors), and lower esophagus (32–40 cm from the incisors). There was no statistically significant difference in lesion location distribution between the two groups (*P* = 0.75), with both groups predominantly having lesions in the upper esophagus.

#### Size

Lesions were divided into two groups based on a maximum diameter of 10 mm: ≤ 10 mm and > 10 mm. There was no statistically significant difference in lesion size distribution between the two groups (*P* = 0.500). The vast majority of lesions (78/88, 88.64%) had a maximum diameter ≤ 10 mm, indicating that most tumors were small in size.

#### Layer of origin

Based on EUS evaluation, there was no statistically significant difference in the distribution of lesion origin layers between the two groups (*P* = 0.374). Most lesions (74/82, 90.2%) originated from the muscularis mucosae, indicating that the majority were superficial in origin. The detailed distribution is shown in Table 2.

**Table 2:** Comparison of lesion characteristics of esophageal submucosal tumors between the two groups

Characteristic	Closure group (*n* = 54)^a^	Non-closure group (*n* = 34)^b^	*P*-value
Lesion location			
Upper esophagus	29 (53.70%)	19 (55.88%)	
Middle esophagus	13 (24.07%)	6 (17.65%)	0.75
Lower esophagus	12 (22.22%)	9 (26.47%)	
Maximum diameter			
≤ 10 mm	49 (90.74%)	29(85.29%)	0.500
> 10 mm	5 (9.26%)	5 (14.71%)	
Layer of origin			
Muscularis mucosae	44 (81.48%)	30 (88.24%)	
Deep mucosal layer	3 (5.56%)	1 (2.94%)	0.374
Submucosal layer	1 (1.85%)	2(5.88%)	
Muscularis propria	0 (0.0%)	1 (2.94%)	

ab: Some patients had multiple lesions, so the number of lesions exceeds the number of patients. Some lesions did not undergo preoperative endoscopic ultrasound examination

### Pathological results

All postoperative specimens were sent for pathological examination. There was no statistically significant difference in the distribution of pathological types between the two groups (*P* = 0.45). Leiomyoma was the predominant pathological type, accounting for70.6% (24/34) of cases in the closure group and 66.7% (36/54) in the non-closure group.

### Clinical outcomes

#### Postoperative diet status

All patients remained nil by mouth on the day of EMR, regardless of postoperative mucosal defect closure status. A clear liquid diet was initiated on the first postoperative day for all patients.

#### Postoperative treatment regimen

The two groups received identical postoperative treatment regimens. All patients underwent proton pump inhibitor (PPI) therapy after the procedure, with no differences based on wound closure status.

#### Postoperative complications

The incidence of postoperative bleeding and perforation was extremely low. In the closure group, one patient (1.9%) experienced minor hematemesis, which improved after conservative treatment; no bleeding events occurred in the non-closure group. There was no statistically significant difference between the two groups in the incidence of postoperative bleeding (*P* = 1.0) or perforation (*P* = 1.0). The bleeding case that occurred in the clip closure group was delayed postoperative bleeding. For the patient with postoperative bleeding in the closure group, oral Yunnan Baiyao capsules were given as conservative hemostatic therapy.

### Hospital stay and costs

The average hospital stay in the closure group and non-closure group was 3.98 ± 1.18 days and 4.47 ± 1.19 days, respectively, with no statistically significant difference (*P* = 0.07). In terms of hospitalization costs, the average cost in the non-closure group (9180.34 ± 2647.80 yuan) was lower than that in the closure group (9745.08 ± 3123.66 yuan). The difference did not reach statistical significance (*P* = 0.397) (Table 3).

**Table 3:** Comparison of hospital stay and costs between the two groups

Characteristic	Closure group (*n* = 52)	Non-closure group (*n* = 32)	*P*-value
Hospital stay (mean ± SD)	3.98 ± 1.18	4.47 ± 1.19	0.07
Costs (mean ± SD)	9745.08 ± 3123.66	9180.34 ± 2647.80	0.397

## Discussion

This retrospective study compared clinical outcomes between active wound closure and non-closure following EMR. There were no statistically significant differences between the two groups in terms of baseline characteristics, lesion features, incidence of immediate postoperative complications (such as bleeding or perforation), or length of hospital stay. These findings suggest that for selected esophageal SMTs, omitting routine wound closure after EMR may represent a safe and effective management strategy. Notably, the non-closure group showed a trend toward lower hospitalization costs, a finding that may have implications for future treatment decisions and economic evaluations.

### Exploring the potential value and indications for active closure

Traditionally, the primary goal of active wound closure (e.g., using metal clips) after EMR has been to prevent delayed bleeding and perforation, particularly in cases involving larger or deeper defects [17, 19, 20]_._ In the present study, the absence of a significant difference in complication rates between the two groups may be explained by the fact that most lesions were small (≤ 10 mm) and predominantly confined to the relatively superficial muscularis mucosae layer. Previous studies have similarly indicated that for low-risk, small wounds, natural processes of coagulation and epithelialization are sufficient to ensure safe healing [19]. Thus, our findings support the notion that not all post-EMR defects require mechanical closure. Moving forward, identifying truly high-risk wounds that warrant intervention—such as those with larger diameters, located in richly vascularized areas, or associated with significant intraoperative bleeding—will be essential for optimizing clinical pathways and advancing precision treatment.

### Safety and cost-effectiveness analysis of the non-closure strategy

In this study, no cases of postoperative bleeding or perforation were observed in the non-closure group, indicating a safety profile comparable to that of the closure group. These findings further support the feasibility of a selective non-closure strategy in appropriate clinical contexts. Notably, although the difference in total hospitalization costs between the two groups did not reach statistical significance, a consistent trend toward lower costs was evident in the non-closure group. This reduction was primarily driven by savings on closure devices such as metal clips. In the context of increasing constraints on medical resources and the ongoing implementation of Diagnosis-Related Group (DRG) and Diagnosis-Intervention Packet (DIP) payment reforms, such a cost-effective strategy holds particular relevance. It offers the potential to reduce patients’ financial burden and alleviate pressure on healthcare expenditures without compromising patient safety—aligning with the core principles of value-based healthcare.

### Limitations of this study

First, as a single-center retrospective design, it is susceptible to selection bias; for instance, physicians may have been more inclined to close lesions perceived as high risk. Second, the sample size—particularly in the non-closure group—is relatively limited. This may render the study underpowered to detect statistically significant differences in rare but clinically significant complications, such as delayed major bleeding. Third, hospitalization costs are subject to the influence of various non-medical factors; therefore, the cost analysis presented should be regarded as exploratory and descriptive. Finally, this investigation focused primarily on short-term outcomes during the index hospitalization. Long-term endpoints, including wound healing quality, local recurrence, and delayed stenosis, were not assessed due to the lack of follow-up data.

### Implications for future research and clinical practice

This study provides preliminary evidence to inform individualized decision-making regarding post-EMR wound management. Future research should focus on the following priorities:

1. Large-scale, multicenter prospective randomized controlled trials are needed to validate the equivalence and safety of the non-closure strategy and to generate higher-quality clinical evidence. 2. Development and validation of a risk prediction model incorporating factors such as lesion size, location, depth, and intraoperative bleeding to better identify patients who may truly benefit from active closure. 3. Long-term follow-up studies to evaluate the effects of both strategies on scar formation, local recurrence, patient-reported outcomes, and overall quality of life. 4. Comprehensive health economic evaluations to more precisely assess the cost-effectiveness of the two approaches.

## Conclusion

In summary, this study demonstrates that for the postoperative management of esophageal SMTs following EMR—under strict patient selection criteria, including small lesion size and superficial origin—both active closure and non-closure of the mucosal defect achieve comparable short-term safety outcomes. Moreover, the non-closure strategy may offer potential advantages in reducing medical costs. We recommend that clinicians adopt individualized wound management strategies based on a comprehensive evaluation of lesion characteristics and patient condition, thereby ensuring medical safety while optimizing healthcare resource utilization.
